# Probation of the offender with high functioning autistic traits and comorbidity. A case study

**DOI:** 10.1192/j.eurpsy.2021.1010

**Published:** 2021-08-13

**Authors:** A. Riolo, R. Keller, R. Battaglia, U. Albert

**Affiliations:** 1 Department Of Mental Health, ASUGI, Trieste, Italy; 2 Department Of Mental Health, ASL Città di Torino, Torino, Italy; 3 Civil Court, Court of Trieste, Trieste, Italy; 4 Department Of Mental Health, University of Trieste, Trieste, Italy

**Keywords:** forensic psychiatry, autism, offender

## Abstract

**Introduction:**

When the Criminal Court Judge applies probation, the offender is entrusted to social assistants for the necessary observation, treatment and support. This case study examines the probation of a young man with high-functioning autistic traits, personality disorder and legal/illegal substance abuse. This young man, who arrived only in adulthood to a diagnosis of autistic traits, is aware only that is non-neurotypical. He does not recognize that he needs treatment for personality disorder, alcohol, substance and drug abuse. He faces a sentence of more than three years in prison but the Judge suspends the criminal trial.

**Objectives:**

Clarify the relationship between high functioning autistic traits, comorbidity with personality disorder and drugs/substance abuse, and crimes committed; also describe the orientation of the Judge and what difficulties arise during the probation.

**Methods:**

Examination of the criminal file and medical documents of the offender, known by social and health services.

**Results:**

The offender correlates the crimes and its frailty with autism and not with antisocial behaviours to gain economic benefits from drug dealing.

**Conclusions:**

The deficit in the social communication and lack of empathy for child victimes, for example, limits the effectiveness of probation. The probation, for a young with high-functioning autistic traits and comorbidity, does not seem to give satisfactory results in terms of rehabilitation and social integration, nor does it produce the extinction of crime.

